# Predictors for low TAVI-prosthesis position assessed by fusion imaging of pre- and post-procedural CT angiography

**DOI:** 10.1007/s00392-020-01654-5

**Published:** 2020-05-12

**Authors:** Philipp Breitbart, Gregor Pache, Jan Minners, Manuel Hein, Holger Schröfel, Franz-Josef Neumann, Philipp Ruile

**Affiliations:** 1 Division of Cardiology and Angiology II, University Heart Center Freiburg-Bad Krozingen, Südring 15, 79189 Bad Krozingen, Germany; 2 Radiology Hegau Bodensee, Practice for Diagnostic Radiology, Singen, Germany; 3 Division of Cardiovascular Surgery, University Heart Center Freiburg-Bad Krozingen, Bad Krozingen, Germany

**Keywords:** TAVI, Computed tomography angiography, Post-TAVI CTA, Fusion imaging, THV positioning, Device landing zone calcification

## Abstract

**Background:**

Low prosthesis position after transcatheter aortic valve implantation (TAVI) is associated with higher rates of new onset conduction disturbances and permanent pacemaker implantations. Purpose of this study was to investigate possible predictors of a low prosthesis position of the SAPIEN 3 (Edwards Lifesciences, Irvine, California, USA) valve type using fusion imaging of pre- and post-procedural computed tomography angiography (CTA).

**Methods:**

CTA fusion imaging was performed in 120 TAVI-patients with 3D-reconstruction of the transcatheter heart valve (THV) position within the device landing zone. A low implantation position was defined according to the manufacturer’s recommendations as > 30% of the prosthesis below the native annulus plane.

**Results:**

A low THV position was found in 17 patients (14%). Patients with low THV position had less calcification of the annulus region and a smaller annulus size compared to patients with a normal or high THV position (*P* = 0.003 and 0.041, respectively). The only independent predictor of a low THV position in multivariate logistic regression analysis was the extent of calcification of the cusp region (odds ratio [CI] 0.842 [0.727–0.976], *P* = 0.022).

**Conclusions:**

Fusion imaging of pre-and post-procedural CTA identified reduced calcification of the cusp region as an independent predictor of a low THV position of the SAPIEN 3. This should be considered when planning the TAVI procedure.

**Graphic abstract:**

Correlation of cusp region calcification and prosthesis position after TAVI

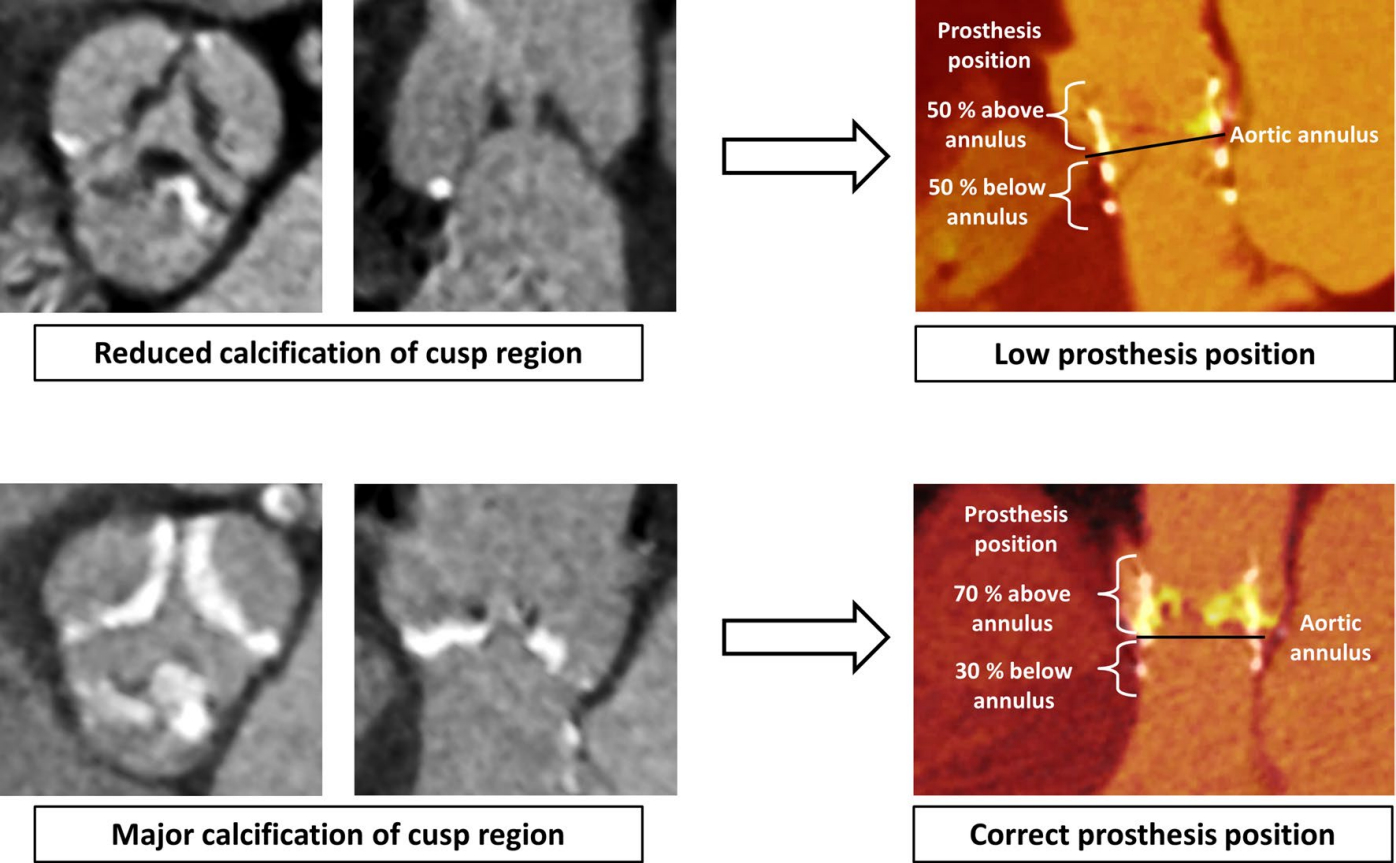

## Introduction

Permanent pacemaker implantations, due to new onset conduction disturbances (CD), are a significant complication after transcatheter aortic valve implantation (TAVI) and have been reported in between 12 and 16% of patients receiving an SAPIEN 3 (Edwards Lifesciences, Irvine, California, USA) transcatheter heart valve (THV) [1–4]. Besides possible effects on long-term outcomes, this complication carries a substantial influence on health economics considering the steadily growing number of TAVI-procedures worldwide, which is even performable with a high success rate in patients about 90 years [5–7]. Prior studies using peri-interventional fluoroscopy identified a low implantation depth of the THV as a predictor for the occurrence of new CD after TAVI [8, 9]. This was confirmed by a recent study from our group using a new fusion technique of pre- and post-procedural computed tomography angiography (CTA) which allows for a three dimensional analysis of the prosthesis position [10]. Limited information is available regarding the impact of structural- and/or procedure-related factors on a low stent position, which may guide a more individualized treatment strategy.

Therefore, the objective of this study was to investigate potential structural- and procedure-related predictors for a low prosthesis position of the SAPIEN 3 THV using a recently developed fusion imaging technique based on fusion of pre- and post-TAVI CTA [10].

## Methods

### Study population

For this retrospective study, we included all patients with evaluable pre-and post-TAVI CTA who had received an SAPIEN 3 between December 2014 and February 2016. In our institution, routine post-TAVI CTAs were performed in all eligible patients. Reasons for not performing a post-procedural CTA were renal insufficiency, frailty and others, as described previously [11]. In all patients, a multidisciplinary, institutional heart team decided on eligibility for TAVI, procedural feasibility, the preferred access route, prosthesis type and size [12]. Valves were implanted either via a transfemoral or transapical access in accordance with well-described standard techniques [13]. Each of the treating interventionalists had an experience of at least 100 or more TAVI procedures. All patients gave their written informed consent for the anonymized use of clinical, procedural, and follow-up data. The study was approved by the local institutional review board.

### Role of the sponsor

Edwards Lifesciences (Irvine, California, USA) sponsored the study, but had no influence on data acquisition and interpretation or writing of the manuscript.

### Image acquisition and analysis

The retrospective ECG-gated pre- and post-TAVI CTAs were performed with a second-generation dual-source CT scanner (Somatom Definition Flash, Siemens Healthcare, Forchheim, Germany) prior to discharge. For contrast-enhanced data acquisition, we used iodinated contrast agent (Imeron 400, Bracco, Konstanz, Germany), with 70 mL for pre- and 50 mL for post-TAVI CTA. The scan was started by means of bolus tracking (region of interest in the left atrium). Images were reconstructed at 50 ms steps throughout the cardiac cycle with a slice thickness of 1 mm and an increment of 0.8 mm (with a stent specific reconstruction kernel B46f for post-TAVI CTA). The detailed protocol for the retrospective ECG-gated cardiac-CTA was described previously [11].

**Fig. 1:**
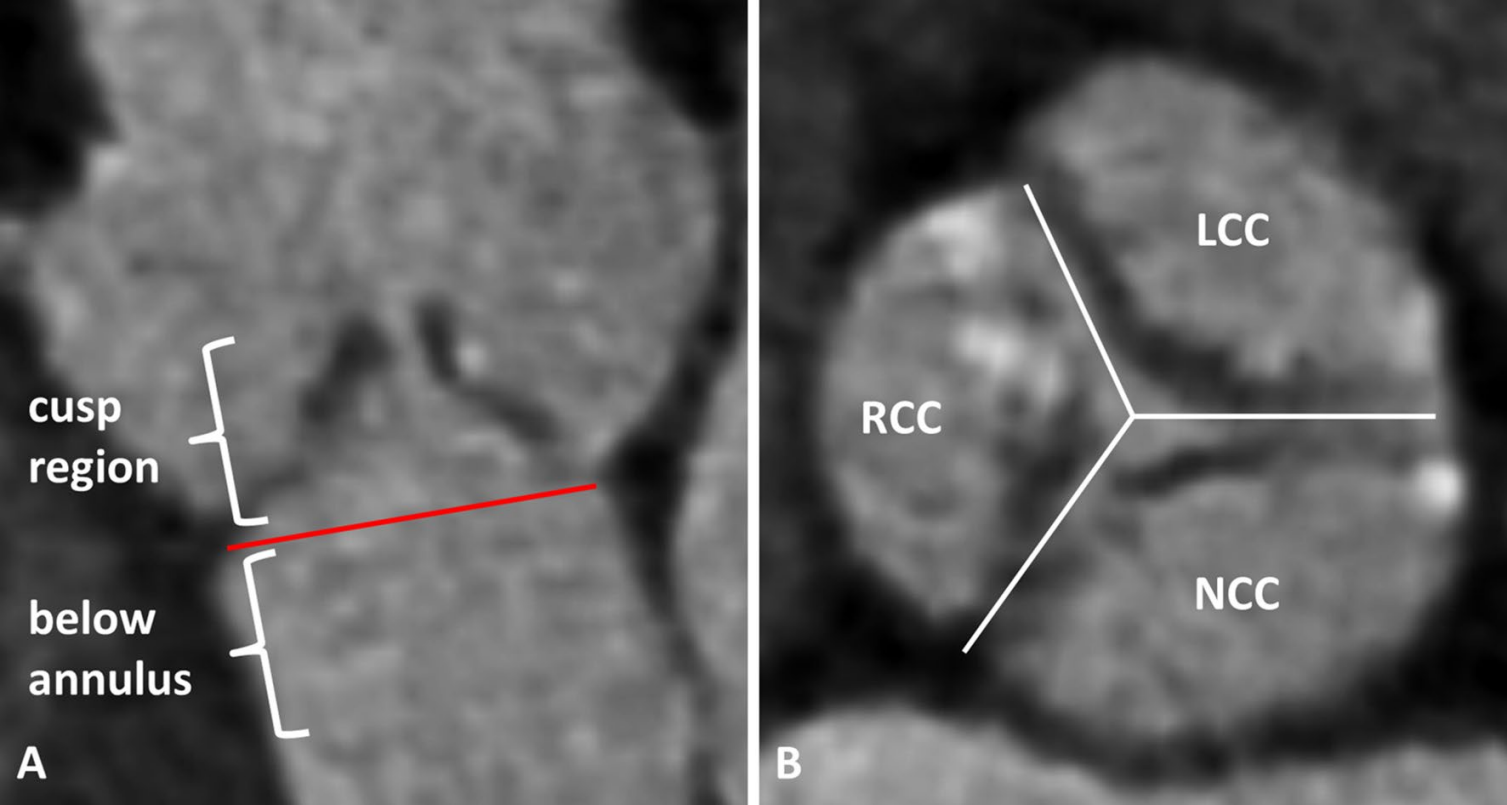
Calcification assessment of the device landing zone. Pre-TAVI contrast-enhanced CTA sagittal oblique and axial reconstruction delineating the two different heights of the device landing zone (**a** red line delineating the annulus plane) and the division of the aortic valve into three cups for the semiquantitative calcification scoring (**b**)

**Fig. 2:**
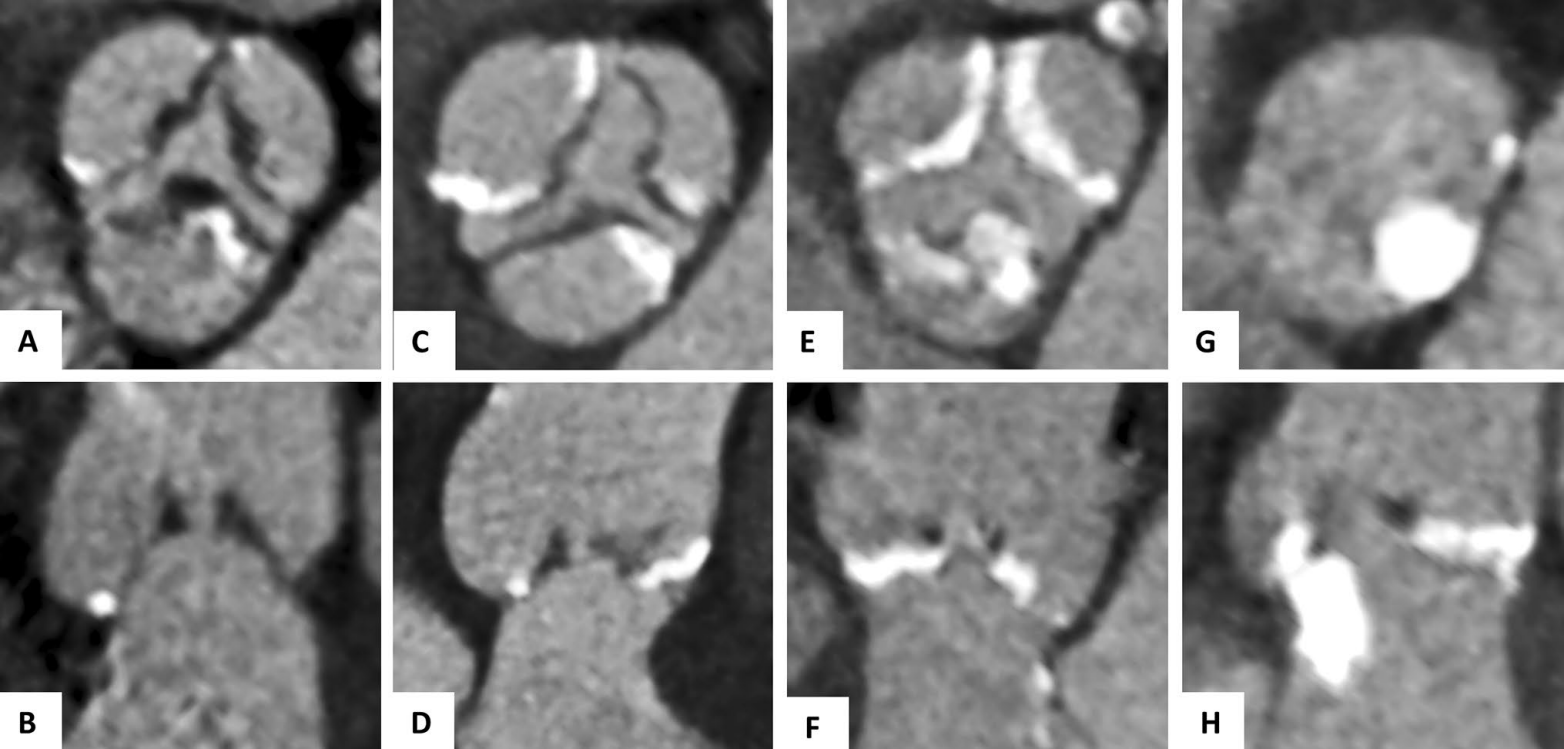
Semiquantitatitve calcification assessment of the device landing zone. Pre-TAVI contrast-enhanced CTA axial and sagittal oblique reconstruction for visual calcification assessment with examples for mild (**a**, **b**), moderate (**c**, **d**) or severe (**e**, **f**) calcification of the cups regions and a severe calcification of the region below the annulus (**g**, **h**)

All image analyses were performed by three experienced readers (G.P., P.R., and P.B.) on a dedicated post-processing workstation (Syngo Multimodality Workplace, Siemens Healthcare, Forchheim, Germany) using multiplanar reformations. The following measurements were conducted based on pre-TAVI CTA:

The aortic annulus area and the area-derived diameter were assessed during systole (as described previously) and the annulus eccentricity was calculated as largest diameter/smallest diameter [14]. We calculated oversizing (%) as (manufacturer reported THV area/annulus area − 1) × 100. The calcification of the device landing zone was semiquantitatively assessed for each cusp (grade 0: no calcification, grade 1: mild calcification as small calcified spots with minimal diameter ≤ 2 mm, grade 2: moderate calcification as calcified spots with minimal diameter more than 2 mm, grade 3: severe calcification as large calcified formations more than 5 mm minimal diameter) and at two distinct heights (cusp region and below annulus, Figs. 1 and 2).

### Fusion imaging

We performed fusion imaging of pre- and post-procedural CTA as previously described [9]. In brief, after delineation of the native annulus plane within the pre-TAVI CTA, the pre- and post-TAVI images were semi-automatically merged at the corresponding reconstruction time point in systole. Thereafter, the fused images were manually adapted to reach optimal alignment of the device landing zone and adjacent structures. Stent length in mm below (= implantation depth) and above annulus as well as the total stent length were measured next to the non-coronary cusp (NCC), left coronary cusp (LCC), and right coronary cusp (RCC). Height variance (%) describes the differences in stent length (above and below annulus) between the three measuring points (NCC, LCC, and RCC) and was calculated as: [maximum stent length in mm (at NCC, LCC or RCC)/minimum stent length in mm (at NCC, LCC or RCC) − 1] × 100.

According to the manufacture’s recommendation, a low prosthesis position (LP) was defined as more than 30 percent of the total prosthesis length below the native annulus plane in the left ventricular outflow tract (LVOT). The corresponding values for a normal position were 20–30% and for a high position, less than 20% of the total prosthesis length below the annulus plane. The two latter groups were merged and served as control.

THV stent area was assessed at three different heights: within the LVOT (entry), mid of the stent and at the aortic edge (exit). The asymmetrical THV expansion (%) specifies the variances of the stent center area compared to the average of the stent entry and exit. It was calculated as [(area stent entry + area stent exit)/(2 × area stent center) − 1] × 100.

### Fluoroscopy projections

**Fig. 3:**
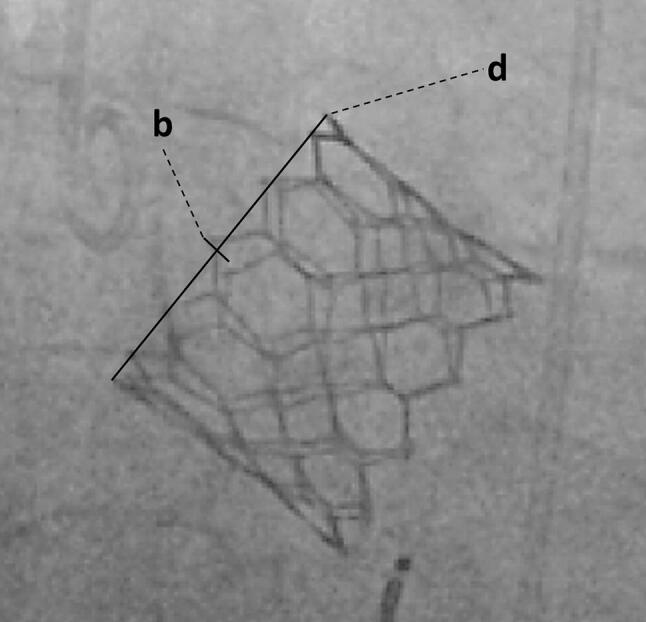
Assessment of post-implantation parallaxes. Post-implantation fluoroscopy image for measurement of the projection-related difference between the anterior and posterior upper prosthesis edge (*b*) in relation to the prosthesis diameter (*d*) to determine possible parallaxes of the THV as an expression of the projection accuracy

To validate the accuracy of the fluoroscopy projection and evaluate its influence on our results, we determined the possible post-implantation parallaxes of the THV. Therefore, we measured the projection-related difference between the anterior and posterior upper prosthesis edge (*b*) in relation to the prosthesis diameter (*d*) and calculated the parallaxes (*α*) as: sin(*α*) = *b*/*d* (Fig. 3).

### Statistical analysis

We performed all statistical analysis with SPSS software (SPSS version 23.0, SPSS, Chicago, Illinois). Categorical data are listed as frequencies and percentages, continuous variables as mean and median with standard deviation or interquartile range, respectively. Differences between groups were examined with the *χ*^2^ test (for categorical variables), Student’s *t* test (for normal distributed continuous variables) or Mann–Whitney *U* Test (non-normal distributed continuous variables). Kolmorogov–Smirnoff test was used to test continuous variables for normal distribution.

Subsequently, we calculated univariate and multivariate logistic regression models for the assessment of the association between potential structural- and procedure-related variables and a low THV position. The multivariate models comprised variables with a *P* value < 0.05 in univariate analysis. Results are presented as odds ratio (OR) with 95% confidence interval (CI). A value of *P* < 0.05 was considered statistically significant.

## Results

Out of 127 patients with pre-and post-TAVI CTA, we performed fusion imaging in 120 patients with evaluable image quality (mean age 83.0 ± 4.5 years, 48.3% female, mean logistic Euroscore 14.2 ± 11.2%). Baseline characteristics are presented in Table 1. The mean annulus area of the overall cohort was 464.2 ± 74.7 mm [2], implanted prosthesis sizes were 23 mm (40.0%), 26 mm (47.5%), and 29 mm (12.5%) with a mean oversizing of 5.8 ± 7.2%. 11.7% of all patients had an implanted permanent pacemaker.

**Table 1:** Baseline and procedural characteristics of the entire study population, the control group and in patients with a low prosthesis position

	All patients (*n* = 120)	Control group (*n* = 103)	Low prosthesis position (*n* = 17)	*P* value
Age (years)	83.0 ± 4.5	83.1 ± 4.4	82.2 ± 5.3	0.400
Female	58 (48.3)	49 (47.6)	9 (52.9)	0.682
BMI (kg/m^2^)	26.8 ± 4.3	27.1 ± 4.4	25.2 ± 3.6	0.115
Logistic euroscore (%)	14.2 ± 11.2	14.3 ± 11.8	13.8 ± 6.5	0.461
Previous pacemaker	14 (11.7)	10 (9.7)	4 (23.5)	0.100
Aortic valve area (cm^2^)	0.76 ± 0.16	0.75 ± 0.16	0.77 ± 0.16	0.795
Annulus area (CTA) (mm^2^)	464.2 ± 74.7	469.9 ± 76.7	429.6 ± 49.6	0.041
Grade of calcification of the cusp region				
Total	4.1 ± 1.1	4.2 ± 1.0	3.4 ± 1.1	0.003
Left coronary cusp	1.2 ± 0.5	1.2 ± 0.5	1.0 ± 0.5	0.043
Right coronary cusp	1.3 ± 0.5	1.4 ± 0.5	1.0 ± 0.5	0.017
Non-coronary cusp	1.6 ± 0.5	1.6 ± 0.4	1.4 ± 0.5	0.055
Grade of calcification of below annulus plane				
Total	0.3 ± 0.5	0.3 ± 0.5	0.4 ± 0.8	0.667
Left coronary cusp	0.1 ± 0.3	0.1 ± 0.2	0.3 ± 0.5	0.345
Right coronary cusp	0.1 ± 0.2	0.1 ± 0.2	0.0 ± 0.0	0.073
Non-coronary cusp	0.2 ± 0.3	0.2 ± 0.3	0.1 ± 0.2	0.359
Access route				0.562
Transfemoral	118 (98.3)	101 (98.1)	17 (100)	
Transapical	2 (1.7)	2 (1.9)	0 (0)	
Prosthesis size				0.191
23 mm	48 (40.0)	39 (37.9)	9 (52.9)	
26 mm	57 (47.5)	49 (47.6)	8 (47.1)	
29 mm	15 (12.5)	15 (14.6)	0 (0.0)	
Postdilatation	17 (14.2)	13 (12.6)	4 (23.5)	0.232
Underfilling	12 (10.0)	10 (9.7)	2 (11.8)	0.793
THV oversizing (%)	5.8 ± 7.2	5.6 ± 7.3	7.1 ± 7.3	0.429
Height variance (%)	5.5 ± 3.9	5.7 ± 4.0	4.4 ± 3.1	0.295
Asymmetrical THV expansion (%)	5.3 ± 2.4	5.3 ± 2.5	4.9 ± 2.1	0.657
New CD post-TAVI (%)	35 (29.2)	26 (25.2)	9 (52.3)	0.020
Post-implantation parallaxes (°)	2.3 [0;3.4]	2.3 [0;3.4]	2.3 [0;3.2]	0.973

Values are mean ± SD, median [interquartile range] or *n* (%)

*BMI* body mass index, *CD* conduction disturbances, *CTA* computed tomography angiography, *TAVI* transcatheter aortic valve implantation, *THV* transcatheter heart valve

### THV position

**Fig. 4:**
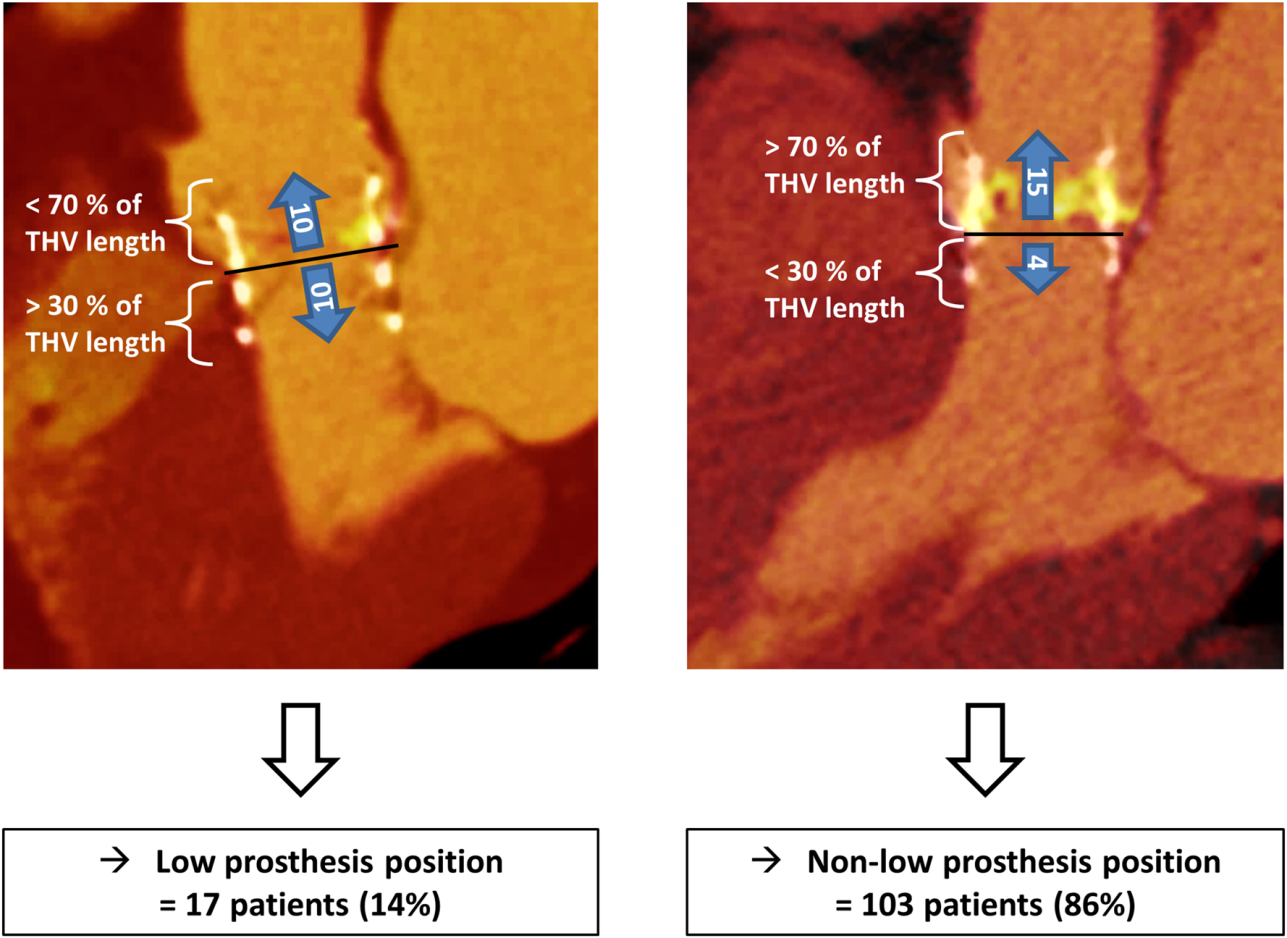
Evaluation of the prosthesis position after TAVI. Fusion images of pre- and post-TAVI CTA of a 80-year-old woman with a low prosthesis position after TAVI (**a**) and a 82-year-old man with a normal prosthesis position, according to the manufacturer’s recommendations (**b**). The numbers within the arrows delineating the mean THV length (in mm) above and below the native annulus plane (= black line)

The mean implantation depth of the THV in the whole cohort was 3.9 ± 2.3 mm below the annulus plane. A LP with more than 30% of the THV below the native annulus plane was detected in 17 patients (14%), whereas the THV was located in a normal or high position (= control group) in 103 patients (86%, Fig. 4).

A comparison of baseline characteristics between patients with a LP and the control group is presented in Table 1. In patients with LP, we observed a smaller mean annulus area [429.6 ± 49.6 vs. 469.9 ± 76.7 mm, *P* = 0.041] and a lower grade of calcification of the cusp region (3.4 ± 1.1 vs. 4.2 ± 1.0, *P* = 0.003), most pronounced in the area of the right coronary cusp (1.0 ± 0.5 vs. 1.4 ± 0.5, *P* = 0.017). Prosthesis size and oversizing or an asymmetrical THV expansion were not different between groups (*P* = 0.191, 0.429, 0.657, respectively). Furthermore, the post-implantation parallaxes of the prostheses showed no influence on implantation depth (median of 2.3° in both groups, *P* = 0.973).

In patients with a LP, we observed more new occurred conduction disturbances post-TAVI (52.3% vs. 25.2%, *P* = 0.020), resulting in more permanent pacemaker implantations (41.2% vs. 11.7%, *P* = 0.002).

### Predictors of a low prosthesis position

In univariate logistic regression analysis, the annulus area and the total grade of calcification of the cusp region were predictors for a LP after TAVI-procedure. After multivariate adjustment, the only independent predictor of LP was reduced calcification of the cusp region (odds ratio [CI] 0.842[0.727–0.976], *P* = 0.022) (Table 2).

**Table 2:** Univariate and multivariate logistic regression model analyses of predictors of a low prosthesis position after TAVI

	Univariate		Multivariate	
	*P* value	Odds ratio [95% confidence interval]	*P* value	Odds ratio [95% confidence interval]
Annulus area	0.047	0.721 [0.522–0.995]	0.249	0.812 [0.570–1.157]
Grade of calcification of the cusp region—total	0.005	0.818 [0.711–0.941]	0.022	0.842 [0.727–0.976]

## Discussion

To the best of our knowledge, this is the first study investigating the influence of structural- (regarding the naïve aortic valve region) and procedural-related criteria on prosthesis position after TAVI. Based on fusion imaging of pre- and post-TAVI CTA, we identified a reduced calcium burden within the cusp region as the sole independent predictor for a low position of the SAPIEN 3.

### Predictors of a low THV position

New conduction disturbances potentially resulting in permanent pacemaker implantation are among the most frequent complication after TAVI [1, 15–17]. Previous studies described a low prosthesis position as an independent risk factor for new conduction disturbances post-TAVI [8–10]. This is in line with our current study findings that patients with a low prosthesis position developed significantly more new conduction disturbances post-TAVI (52 vs. 25%). To the best of our knowledge, there are no studies published investigating predictors of a low THV position. Our study identified a small amount of calcification of the cusp region as the sole predictor for a low SAPIEN 3 THV position. Of note, none of the other procedural characteristics or prosthesis-related factors were associated with a low THV position.

### Potential mechanism of the association between low calcium burden and low THV position

According to the manufacture’s recommendation, the center of the THV should be positioned at annulus height before starting valve deployment. During valve expansion, the lower part of the SAPIEN 3 THV shortens twice as much as the upper one. In case of moderate or heavily calcified cusps, a fixation of the prosthesis struts during valve expansion is hampered in this region. Therefore, the THV will lock into position through the upper struts into the mostly softer wall of the aorta ascendens. The subsequent asymmetric THV shortening results in a cranial shift of the lower THV-part during expansion with an optimal final position of 70 percent above annulus. Presumably, a relevant calcification is considered for this process. If the cusp region is insufficiently calcified, the central prosthesis parts can also fixate in the annulus wall at an early stage of THV deployment. Therefore, the cranial shift of the lower prosthesis parts is decreased and the THV will remain in lower position towards the LVOT.

### Clinical implications

Previous studies from our group and others described a severe calcification of the device landing zone as an independent predictor of new CD [10, 18, 19]. It has been speculated that the calcifications are causing the new CDs directly via impingement of the conduction system.

On the other hand, using fusion imaging of pre-and post-procedural CTA, we show here that a low grade of calcification of the cusp region is independently associated with an increased risk of low position of the SAPIEN 3. A low THV position may cause CD due to high contact pressure of deeply implanted stents struts on the conduction system. Thus, the advantage of a low calcium burden with respect to the new pacemaker implantation rate may be lost if a low position of the SAPIEN 3 cannot be avoided. According to the findings of the current study, a low calcium burden should be considered as a predictor of a low THV position when planning the TAVI procedure and may influence the positioning of the SAPIEN 3 before deployment.

## Limitations

One limitation of the study is the restricted sample size of our cohort. Consequently, some alternative predictors, such as pre-existing CD or annulus size and eccentricity, might have been missed. For the same reason, we are unable to assess the relation between low TVH position and the need for new pacemaker implantation.

Moreover, our patients had received only one valve type. It remains unclear, if our results are transferable to other valve types. We performed a semiquantitative calcium analysis rather than calcium scoring according to the Agatston method, since (for radiation reduction reasons) no native CT scans pre-TAVI were available. Nevertheless, this method provided meaningful data to identify a low calcification grade as a predictor for LP.

Since we did not perform post-TAVI CTAs in patients with severe frailty or renal insufficiency, it remains questionable whether our results are transferable to this patient cohort.

## Conclusion

Fusion imaging of pre-and post-procedural CTA identified reduced calcification of the cusp region as an independent predictor of a low THV position of the SAPIEN 3. This should be considered when planning the TAVI procedure.

### Clinical perspectives

In view of the fact, that further increasing numbers of worldwide TAVI procedures are expected, adequate knowledge of the reasons and their prevention for the occurrence of TAVI complications is required. As previously described, a low position of the THV is associated with higher rates of new conduction disturbances after TAVI. Based on this analysis of 120 TAVI-patients with the fusion imaging method, we identified reduced calcification of the cusp region as an independent predictor of a low THV position of the SAPIEN 3. This should be considered when planning the TAVI procedure to reduce the burden of new conduction disturbances mostly leading to pacemaker implantation.

## Abbreviations

CD: Conduction disturbances
CTA: Computed tomography angiography
LP: Low prosthesis position
LVOT: Left ventricular outflow tract
TAVI: Transcatheter aortic valve implantation
THV: Transcatheter heart valve
